# Retracted: Chen, Y, Zhang, W, Kadier, A, Zhang, H, Yao, X. MicroRNA‐769‐5p suppresses cell growth and migration via targeting NUSAP1 in bladder cancer. J Clin Lab Anal. 2020; 34:e23193

**DOI:** 10.1002/jcla.24893

**Published:** 2023-05-14

## Abstract

https://doi.org/10.1002/jcla.23193. The above article, published online on 03 January 2020 in Wiley Online Library (https://onlinelibrary.wiley.com), has been retracted by agreement between the authors, the journal's Editor‐in‐Chief, Professor Rong Fu, and Wiley Periodicals, LLC. The retraction has been agreed following concerns raised by a third party about image duplication (Figure 3H with Figure 2C from https://doi.org/10.18632/aging.103092 and Figure 3C from https://doi.org/10.1016/j.biopha.2018.03.173; Figure 2J with Figure 7D from https://doi.org/10.18632/aging.202847; Figures 2G,I and 4C,F, with Figures 2I,J, 3I,J, 5D, 6D and 7F from https://doi.org/10.3389/fonc.2020.01104, and 5F, 6G, and 7J from https://doi.org/10.18632/aging.103762; Figure 5A with Figure 2C from https://doi.org/10.1042/bsr20191330). Upon investigation, the journal determined that the duplicated images were provided by a third party company who performed the experiments on behalf of the authors. The authors were unable to verify the provenance of the images. As a result, the journal no longer has confidence in the results and conclusions drawn and are issuing a retraction.

https://doi.org/10.1002/jcla.23193. The above article, published online on 03 January 2020 in Wiley Online Library (https://onlinelibrary.wiley.com), has been retracted by agreement between the authors, the journal's Editor‐in‐Chief, Professor Rong Fu, and Wiley Periodicals, LLC. The retraction has been agreed following concerns raised by a third party about image duplication (Figure 3H with Figure 2C from https://doi.org/10.18632/aging.103092 and Figure 3C from https://doi.org/10.1016/j.biopha.2018.03.173; Figure 2J with Figure 7D from https://doi.org/10.18632/aging.202847; Figures 2G,I and 4C,F, with Figures 2I,J, 3I,J, 5D, 6D and 7F from https://doi.org/10.3389/fonc.2020.01104, and 5F, 6G, and 7J from https://doi.org/10.18632/aging.103762; Figure 5A with Figure 2C from https://doi.org/10.1042/bsr20191330). Upon investigation, the journal determined that the duplicated images were provided by a third party company who performed the experiments on behalf of the authors. The authors were unable to verify the provenance of the images. As a result, the journal no longer has confidence in the results and conclusions drawn and are issuing a retraction.

